# Enrichment of Early Fetal-Liver Hemopoietic Stem Cells of the Rat Using Monoclonal Antibodies Against the Transferrin Receptor, Thy-1, and MRC-Oχ82

**DOI:** 10.1155/1995/85036

**Published:** 1995

**Authors:** Kenneth Crook, Simon V. Hunt

**Affiliations:** 1 Dunn School of Pathology University of Oxford South Parks Road Oxford OX1 3RE United Kingdom; 2 MRC Human Genetics unit Western General Hospital Crewe Road Edinburgh EH4 2XU Scotland

**Keywords:** Fetal liver, flow cytometry, lymphopoiesis, MRC-Oχ7, MRC-Oχ26, MRC-Oχ82, pluripotent stem cell

## Abstract

Fetal livers from inbred rat fetuses at 14 days' gestation were dispersed into a single-cell suspension by physical disruption and collagenase digestion. Pluripotent stem cells were characterized and partially purified by a combination of monoclonal antibodies. These included CD71 (anti-transferrin receptor, MRC-Oχ26, used for rosetting), Cdw90 (anti- Thy-1, MRC-Oχ7), and the newly described MRC-Oχ82 (reacting with myeloid cells in peritoneal exudate), employed in FACS sorting. Enrichment was monitored by long-term reconstitution of lethally irradiated congenic rats genetically distinguishable from the donor by an allelomorphic variant of the CD45 cell-surface antigen. At intervals from 3 months to 1 year, lymph-node cells and peritoneal exudate cells were biopsied for analysis by two-color flow cytometry-one color to determine donor origin, the other to identify Th cell (CD4+), Tc cell (CD8+), B cell (sIg+ or CD45RC+ ), neutrophil (Oχ82 + or Oχ43- ), and macrophage (Oχ43+) compartments. The degree of chimaerism was taken as the read out of stem-cell activity. No significant differentials between lymph-node and peritoneal exudate chimaerisms were detected in any of the recipients; therefore, the enrichment procedure revealed only pluripotent cells, not stem cells of restricted potency. All recovered stem-cell activity was in the Oχ26(CD71)-negative, Oχ7(CDw90)-positive, Oχ82-positive fraction. In the optimum case, an enrichment of very roughly 200-fold in cell-for-cell activity was obtained.

Rat bone-marrow colony-forming units in the spleen (CFUs-12) were found to lack the surface antigens recognized by the monoclonal antibodies CD53 (MRC-Oχ44), MRC-Oχ39, MRC-Oχ59, and 144.2.15. These would provide a strategy for their enrichment by depletion.


*Developmental Immunology*, 1996, Vol 4, pp. 235–246
Reprints available directly from the publisher
Photocopying permitted by license only

© 1996 OPA (Overseas Publishers Association)
Amsterdam B.V. Published in The Netherlands
by Harwood Academic Publishers GmbH
Printed in Singapore

### Enrichment of Early Fetal-Liver Hemopoietic Stem Cells of the Rat Using Monoclonal Antibodies Against the Transferrin Receptor, Thy-1, and MRC-OX82

KENNETH CROOK* and SIMON V. HUNT

*Dunn School of Pathology, University of Oxford, South Parks Road, Oxford OX1 3RE, England*

Fetal livers from inbred rat fetuses at 14 days' gestation were dispersed into a single-cell suspension by physical disruption and collagenase digestion. Pluripotent stem cells were characterized and partially purified by a combination of monoclonal antibodies. These included CD71 (anti-transferrin receptor, MRC-OX26, used for rosetting), Cdw90 (anti-Thy-1, MRC-OX7), and the newly described MRC-OX82 (reacting with myeloid cells in peritoneal exudate), employed in FACS sorting. Enrichment was monitored by long-term reconstitution of lethally irradiated congenic rats genetically distinguishable from the donor by an allelomorphic variant of the CD45 cell-surface antigen. At intervals from 3 months to 1 year, lymph-node cells and peritoneal exudate cells were biopsied for analysis by two-color flow cytometry—one color to determine donor origin, the other to identify Th cell (CD4+), Tc cell (CD8+), B cell (sIg+ or CD45RC+), neutrophil (OX82+ or OX43−), and macrophage (OX43+) compartments. The degree of chimaerism was taken as the read out of stem-cell activity. No significant differentials between lymph-node and peritoneal exudate chimaerisms were detected in any of the recipients; therefore, the enrichment procedure revealed only pluripotent cells, not stem cells of restricted potency. All recovered stem-cell activity was in the OX26(CD71)-negative, OX7(CDw90)-positive, OX82-positive fraction. In the optimum case, an enrichment of very roughly 200-fold in cell-for-cell activity was obtained.

Rat bone-marrow colony-forming units in the spleen (CFUs-12) were found to lack the surface antigens recognized by the monoclonal antibodies CD53 (MRC-OX44), MRC-OX39, MRC-OX59, and 144.2.15. These would provide a strategy for their enrichment by depletion.

KEYWORDS: Fetal liver, flow cytometry, lymphopoiesis, MRC-OX7, MRC-OX26, MRC-OX82, pluripotent stem cell

### INTRODUCTION

It has long been known that fetal-liver stem cells differ from adult bone-marrow stem cells (Micklem and Loutit, 1966; Micklem et al., 1972; Hunt and Fowler, 1981). In competitive repopulation experiments, the fetal-liver cells always made the major, and continuously increasing, contribution to the eventual phenotype, even with excess marrow cells (Micklem et al., 1972). Therefore, there may be a surface antigenic distinction between these two cell types, because, whether the potency difference is intrinsic (reflecting a developmentally controlled genetic program) or extrinsic (for instance, homing to a particular microenvironment), it is likely to be manifest in a surface molecular difference. The use of cell-surface phenotype, as defined by monoclonal antibodies, has been the method most exploited to enrich pluripotent hemopoietic stem cells. It has provided them virtually pure in the mouse and human and helped to define stem-cell heterogeneity better (Spangrude et al., 1988; Szilvassy et al., 1989; Andrews et al., 1990; Terstappen et al., 1991; Keller, 1992; Uchida et al., 1993; Spangrude, 1994). This strategy of purification, by comparison with others (e.g. density centrifugation, Jones et al., 1990; or Rh 123 fluorescence, Bertoncello et al., 1991; Srour et al., 1991), may in addition provide clues about the adhesion or signaling characteristics of cell-surface molecules on stem cells.

* Corresponding author. Present address: MRC Human Genetics Unit, Western General Hospital, Crewe Road, Edinburgh EH4 2XU, Scotland.

Here we aimed to enrich stem cells from day-14 rat fetal liver by defining their surface phenotype. In studying the liver at this early stage in organogenesis (about 1 day after it becomes sufficiently erythroid for naked-eye identification in the rat), the hierarchy of stem cells may be simpler than at both later stages such as day 17 studied previously (Hunt, 1979; Jefferies et al., 1985a), and in the adult. At day 14, promyelocytes and prolymphocytes are not yet evident. To define enrichment of stem-cell activity, potency in two respects must be measured: (1) self-renewal and (2) ability to repopulate all hemopoietic compartments. As others have emphasized (Harrison, 1980; Jordon et al., 1990; Keller and Snodgrass, 1990), *in vivo* long-term repopulation assays satisfy these criteria best. We have used a refinement of the Hunt and Fowler (1981) long-term reconstitution assay in irradiated rats, using two strains as cell host and donor that are congenic for an allelomorphic variant of the rat RT7 (CD45 equivalent) surface antigen (Newton et al., 1986). CD45 is expressed on virtually all hemopoietic cells, so that in the various lympho-myeloid subsets, host-type and donor-type progeny can be distinguished by two-color flow cytometry. By determining chimaerism by registering the allelic phenotype of individual cells, rather than that of populations of cells as is necessary for enzyme polymorphic markers (e.g., Harrison, 1980; Flake et al., 1986) or retroviral integration sites (Dick et al., 1985; Lemischka et al., 1986), no assumptions are necessary about the constancy of marker expression per cell. A constant dose of host syngeneic bone-marrow cells is also given to all rats to provide both a source of more mature progenitor cells to ensure the survival of the chimaeras immediately following irradiation, as well as a standard against which the purified stem cells can compete. Here, we have used this assay to measure the reconstitution *in vivo* for intervals between 1 and 12 months in the $CD4^+$, $CD8^+$, and surface $Ig^+$ lymphoid compartments, and among macrophage and neutrophil peritoneal exudate cells.

To characterize surface phenotype, we sought mAb combinations that would identify a small proportion of fetal-liver cells that contained all totipotent stem-cell activity. This would maximize enrichment of activity. We knew that all such activity in collagenase-digested single-cell suspensions of day-17 liver was in the Transferrin-receptor negative ($OX26^- = CD71^-$) (Jefferies et al., 1985a) and Thy-1-positive ($OX7^+ = CDw90^+$) (Hunt, 1979) fraction. One aim of the present paper was to investigate whether this was true also of day-14 liver. In the next step, another mAb was selected from a panel of 36, which were more or less well characterized against rat lympho-hemopoietic cell-surface antigens. This picked out a subset of OX26-negative day-14 liver cells and was also shown to react on "stem cells" when screened in a standard bone-marrow colony-forming-unit assay (fetal liver would have been preferred, but it does not form spleen colonies in rats). The single mAb selected for further analysis of the fetal-liver stem-cell phenotype was MRC-OX82, originally described as staining myeloid cells (Highnam, unpublished). Stem-cell activity in day-14 fetal liver that had been depleted of OX26-reactive cells and then had been sorted by two-color flow cytometry for OX7 and OX82 was measured in long-term repopulation of irradiation chimaeras to determine the degree of enrichment. The lymphoid compartment was sampled in lymph nodes, and the myeloid compartment in peritoneal washes, of the irradiated hosts.

### RESULTS

### Purifying OX26-Negative Fetal-Liver Cells

Figure 1(a) shows the fluorescence profile of OX26 staining day-14 fetal-liver cells, before and after rosetting to deplete the OX26-reactive cells. Typically, 90–95% of fetal liver at this stage was positive for OX26, compared to approximately 20% at day 17, Jefferies et al., 1985a; rosetting gave OX26-negative cells over 90% pure. To measure stem-cell activity in this OX26-negative fraction, various doses of cells before and after rosetting were injection into irradiated rats, and T- and B-cell chimaerisms in peripheral lymph nodes were measured after 8–12 weeks. The results (Fig. 2) show an enrichment of activity of about ten fold. This corresponded to the enrichment determined by flow cytometry, indicating that few stem cells had been lost in the rosetting step. It was not worth assaying the activity in the rosette-containing population because of their contamination by OX26-negatives.

### Phenotype of OX-26-Negative Fetal-Liver Cells

To further define and subdivide the OX26-negative population, a range of monoclonal antibodies was tested for reactivity on these cells. The antibodies

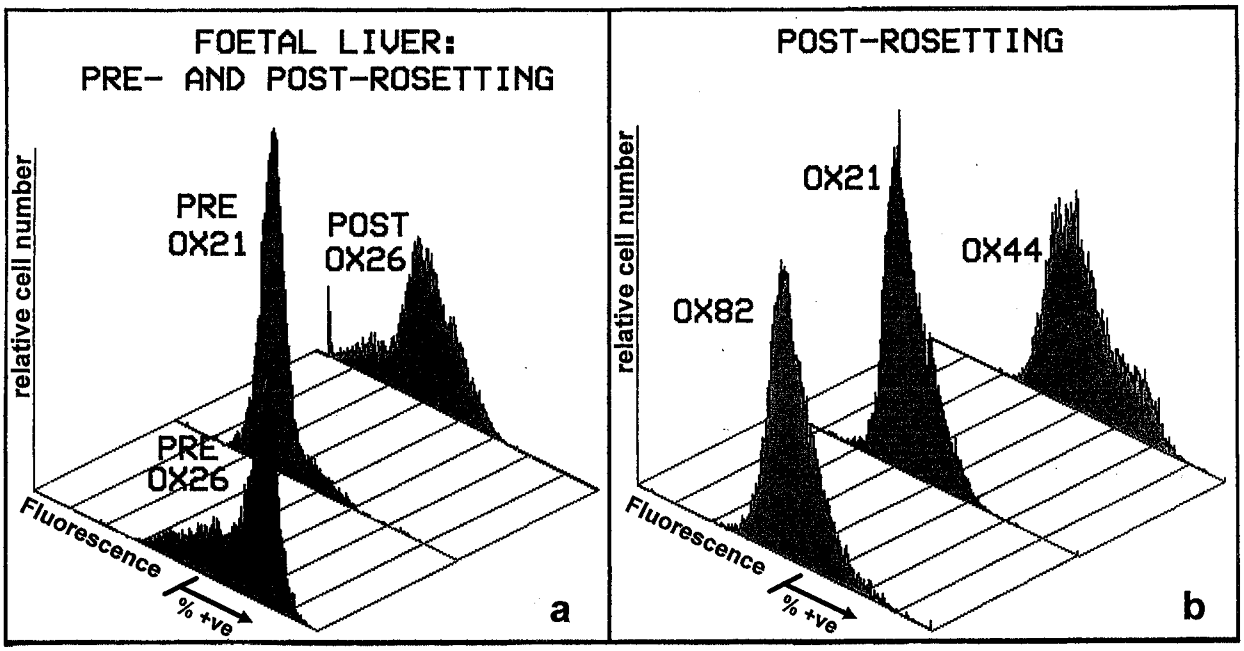

FIGURE 1. (a) Staining of day-14 fetal liver with anti-transferrin-receptor antibody (OX26) and with negative control antibody (OX21) before and after rosette depletion to remove OX26-positive cells. Percent brighter than the marker: Pre-OX26, 78%; pre-OX21, 0.5%; post-OX26, 0.6%. (b) Staining of OX26-depleted day-14 fetal-liver cells with OX82 and OX44 (CD53) antibodies. Percent brighter than the marker: OX44, 16.4%; OX82, 9.1%; OX21, 0.8%. In both panels, the fluorescence intensity is logarithmic.

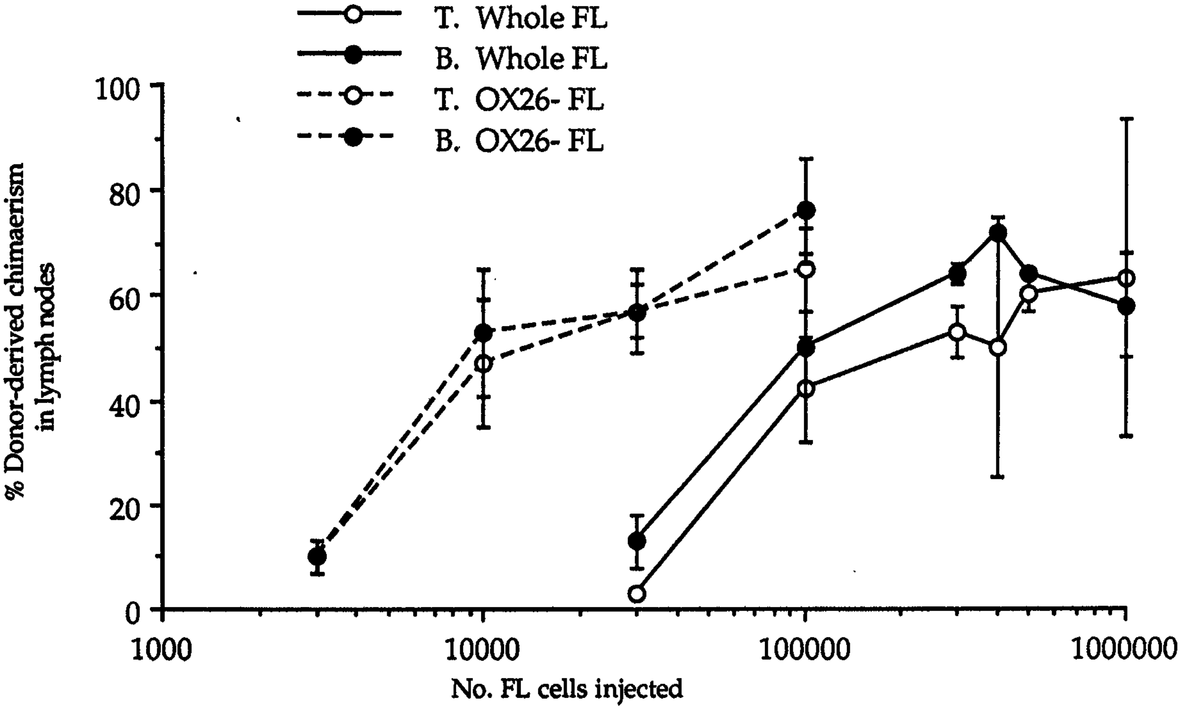

FIGURE 2. Lymphoid reconstitution of irradiated recipient rats by whole and by OX26$^-$ day-14 fetal liver. Donor-derived (RT7$^{b+}$) cells also staining positively for either CD4 or for sIg were measured in cervical lymph nodes 8–12 weeks following injection of FL cells in the standard repopulation assay, competing against 5 x $10^6$ bone-marrow cells. Two to six rats per group. Note the potent activity of $10^5$ unseparated fetal liver cells, and the comparable activity of $10^4$ OX26-negative cells.

could broadly be divided into three categories: those that did not react detectably with OX26$^-$ cells, namely, OX2, OX3 (MHC Class II), W3/25 (CD4), OX8 (CD8), OX34 (CD2), OX32 and OX51 (both CD45RC), OX39 (CD25), OX40, OX42 (CD11b/c); those that reacted with the majority, viz. OX7 (Thy-1), OX45 and OX46 (both CD48), 94–47, UB11, UB12; and those that subdivided the population and would thus be potentially useful for further enrichment of a stem-cell population. These were OX1 (CD45), OX44 (CD53), OX50 (CD44), W3/13 (leukosialin, CD43), OX41, OX43, OX47, OX82, and some other poorly characterized mAbs. Some examples of the fluorescence profiles of each of these mAb categories are shown in Fig. 1(b).

### CFUs Assays

Seven mAbs reacting with subpopulations of OX26-negative fetal liver cells (between 2 and 20% above background) were used in one-color sorts of young adult rat bone marrow for CFUs assay (Table 1). Three types of results were seen: CFUs activity among only the negatives (OX39, OX59, 144.2.15); CFUs activity in the positively staining fractions (OX27, OX82); and instances where CFUs activity was reduced and not fully returned in either the positive or negative fractions (OX44, 94.30). Among the mAbs that did stain CFUs, OX82 was selected for further study on fetal liver. This mAb identifies myeloid cells and stromal elements in a variety of tissues in the adult. Highnam and Hunt (unpublished) have shown that it labels bone-marrow stem cells assayed by the long-term repopulation assay in irradiated hosts. By Western blotting of Brij-96 lysates of bone marrow, it reacts with a molecule about 35 kD, unreduced (data not shown).

### Two-Color FACS Sorts of Fetal Liver for OX26 (Transferrin Receptor) and OX82

Day-14 fetal-liver cell suspensions were rosetted to deplete OX26-bearing cells as before. The depleted population was further exposed to a mixture of biotinylated OX26 with OX82, or, as a control, to biotinylated OX26 with OX21. Phycoerythrin-avidin (red) and FITC rabbit anti-mouse Ig (green) were used as second-stage reagents. Thus, any residual red-stained cells could be gated out by the FACS to give essentially 100% pure OX26-negative cells. Figure 1(b) shows the heterogeneous levels of staining by OX82. OX82-positive and OX82-negative fractions were tested for their ability to restore hemopoiesis in irradiated hosts by the long-term repopulation assay (to beyond 1 year post reconstitution). The T-cell (CD4$^+$), B-cell (sIg$^+$), macrophage (OX43$^+$), and neutrophil (OX82$^+$) compartments were analyzed for chimaerism. Both at 3 months, the lymphoid [Fig. 3(a)] and myeloid [Fig. 3(b)], and at 12 months [Fig. 3(c)], the lymphoid, analyses showed clearly that all the stem-cell activity was returned in the OX26$^-$OX28$^+$ fraction. Over 50% chimaerism was detected from 2700 cells; therefore, their activity could be equated with that of at least $5 \times 10^6$ bone-marrow cells, if we assume that unseparated and separated stem cells have similar reconstitution kinetics. In some case [e.g. in Fig. 3(b), compare OX21 Dim and Whole FL], minor loss of activity was noted, for reasons discussed in Materials and Methods. The level of chimaerism was similar for all compartments, so there was no evidence for the separation of a committed stem cell, even though OX82 recognizes a myeloid-restricted antigen (in the Discussion, the possibility is considered that this procedure simultaneously copurifies two or more committed stem cells rather than one pluripotent stem cell, and is rejected as being unlikely). The control sort with OX21 yielded all activity in the negative fraction, as expected. This whole experiment was repeated twice more, with entirely similar results.

The approximate factor of enrichment of stem-cell activity was derived from a comparison of the number of cells from the initial unsorted population and from the sorted OX26$^-$OX82$^+$ fraction that were able to produce equivalent chimaerisms. In the

TABLE 1
Day-12 CFUs Activity in Bone-Marrow Fractions Sorted by FACS

| mAB | OX39 | OX59 | 144.2.15 | OX44 | 94.30 | OX27 | OX82 |
|---|---|---|---|---|---|---|---|
| Specificity | CD25: IL-2R | Ig | Unknown | CD53 | Unknown | MHC Class I | Unknown |
| CFUs: Dim | 16 ± 6 | 31 ± 6 | 17± 14 | 9 ± 7 | 4.5 ± 4 | 0 | 0.7 ± 0.7 |
| CFUs: Bright | 0.5 ± 0.7 | 0.7 ± 1 | 0.7 ± 0.6 | 1.3 ± 1.3 | 4 ± 2 | 15 | 17.2 ± 4 |

Unseparated bone marrow gave the following CFUs: $10^6$: 21 ± 9.6; 0.5 × $10^6$: 11.1 ± 2.1; 0.1 × $10^6$: 7 ± 4. These data are presented net of background endogenous CFUs (= 1). The number of cells injected was such that Bright + Dim = $10^6$, partitioned according to the FACS-determined percentage in each fraction. Data given as mean ±1 S.D. ($n$ = 1 to 6).

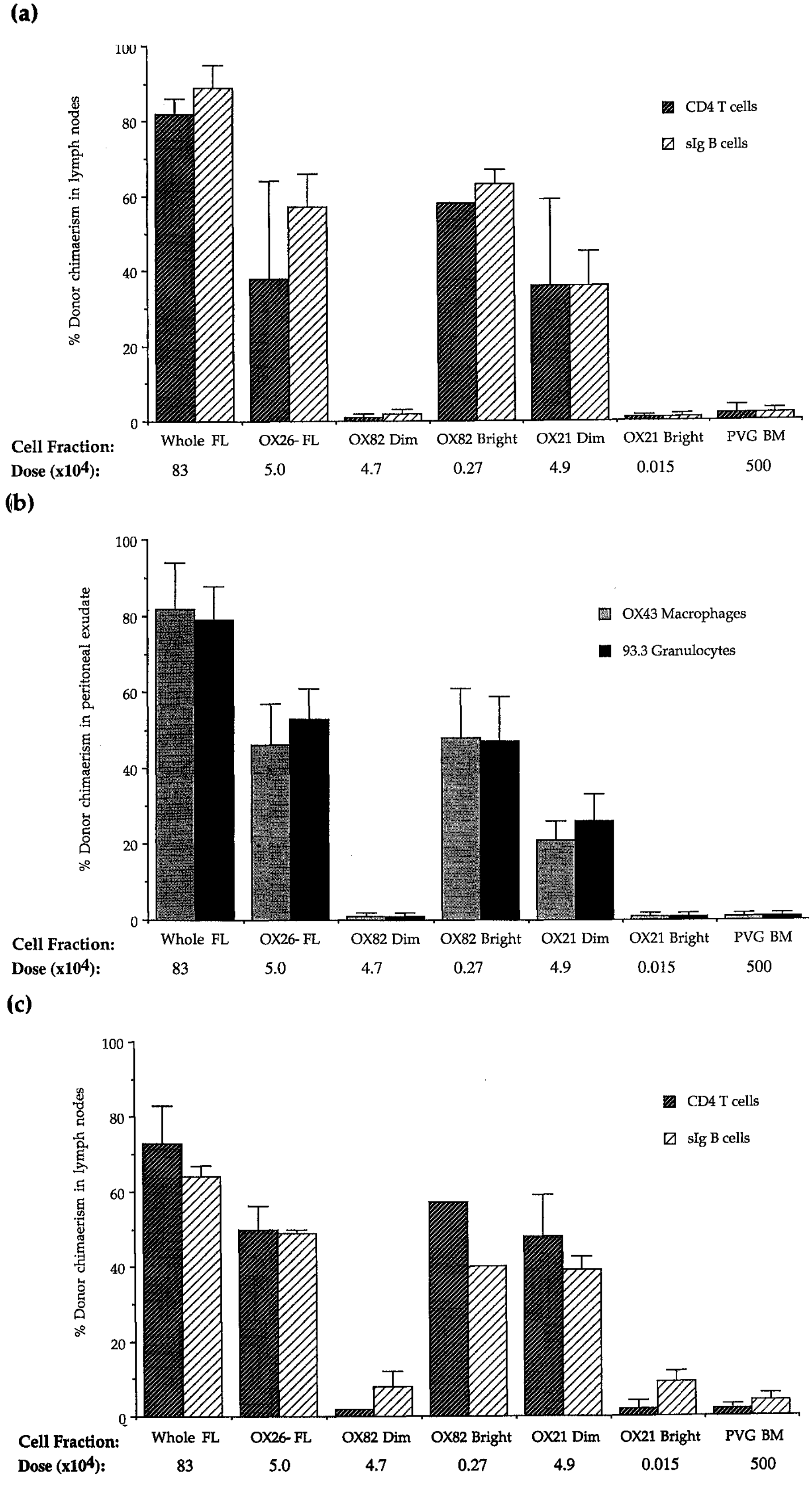

FIGURE 3. Donor-derived chimaerism in peripheral lymph nodes at 3 months following reconstitution with fractions of fetal liver 2-color sorted for OX26 and OX82. T cells were identified by staining with the anti-CD4 mAb W3/25, and B cells by the anti-mIg mAb OX12. "Whole FL" denotes unseparated fetal-liver cells; "OX82 Dim" and "Bright" refer to the cells falling respectively into the negative and positive FACS sorting gates; "OX21 Dim" and "Bright" were the equivalent populations for the OX21 control sort; "OX26$^-$ FL" refers to cells that had been rosette-depleted, but that were not sorted; "PVG BM" refers to rats that received only a dose of $5 \times 10^6$ syngeneic (RT7a) cells, to provide an antibody-staining negative control. The indicated numbers of cells injected for each fraction were calculated as described in Materials and Methods. Error bars represent one standard deviation. One to three rats per group. (b) Donor-derived chimaerism in peritoneal exudate cells at 3 months following reconstitution. Macrophages were defined by staining with the mAb OX43, and neutrophils by staining with the mAb OX82. (c) Donor-derived chimaerism in peripheral lymph nodes at 12 months following reconstitution. Analysis as in Fig. 3(a).

three experiments, the enrichments were 23-fold, 58-fold, and 218-fold. These results confirm that day-14, fetal liver contains long-term repopulating stem cells, all of which lack Transferrin receptor (OX26), and show that OX82 reacts with all such stem cells. We then attempted further enrichments using OX7 (specific for rat Thy-1) because (a) of its well-established stem-cell reactivity (Goldschneider et al., 1978; Hunt, 1979) and (b) pilot experiments showed that it labeled a sub-population of $OX82^+$ OX26, fetal-liver cells.

### Two-Color Sorts for OX82- and OX7-Stained Cells from OX26-Negative Fetal Liver

Day-14 fetal liver cells were rosette-depleted of OX26-reactive cells and prepared for sorting as before except that they were stained with biotinylated OX7 and OX82. Double-positive $OX7^+OX82^+$ cells were separated from the rest. A negative control sort separated cells stained with OX21 for both fluorescein and phycoerythrin using the same gates. Fluorescein contour plots, showing sort gates, of both test and control stained populations for sorting are presented in Fig. 4. In this case, biopsied hemopoietic tissues were analyzed with a modified range of subset-specific mAbs, i.e., OX22 (CD45RC-bright B cells), OX8 (CD8), W3/25 (CD4), and OX43-negative peritoneal exudate (granulocytes). All recovered stem-cell activity, repopulating all these compartments for over 300 days, was found in the $OX7^+OX82^+OX26^-$ population (Fig. 5). In the negative-control sort, no activity was found in the $OX21^+$ fraction, all reconstitution coming from the cells falling in the dull windows (not shown). This experiment was performed twice with similar results. Because the proportion of $OX82^+OX26^-$ cells that stains with OX7 is about 1/2 to 1/3, the extra enrichment obtainable by including OX7 is about two- to threefold. Results from CDw90 staining of rat bone marrow (Goldschneider et al., 1978) suggest that further sorting for the very brightest OX7-staining cells might improve enrichment even further.

### DISCUSSION

In the present work, we have extended the observations of Jefferies et al., (1985a) describing a lack of reactivity of the anti-transferrin receptor monoclonal antibody OX26 on pluripotent stem cells from day-17 rat fetal liver, and those of Hunt (1979) and Highnam (unpublished) showing that OX7 (anti-Thy-1) and OX82 (anti-myeloid) recognize pluripotent stem cells from adult rat bone marrow. By combining these three monoclonal antibodies in a

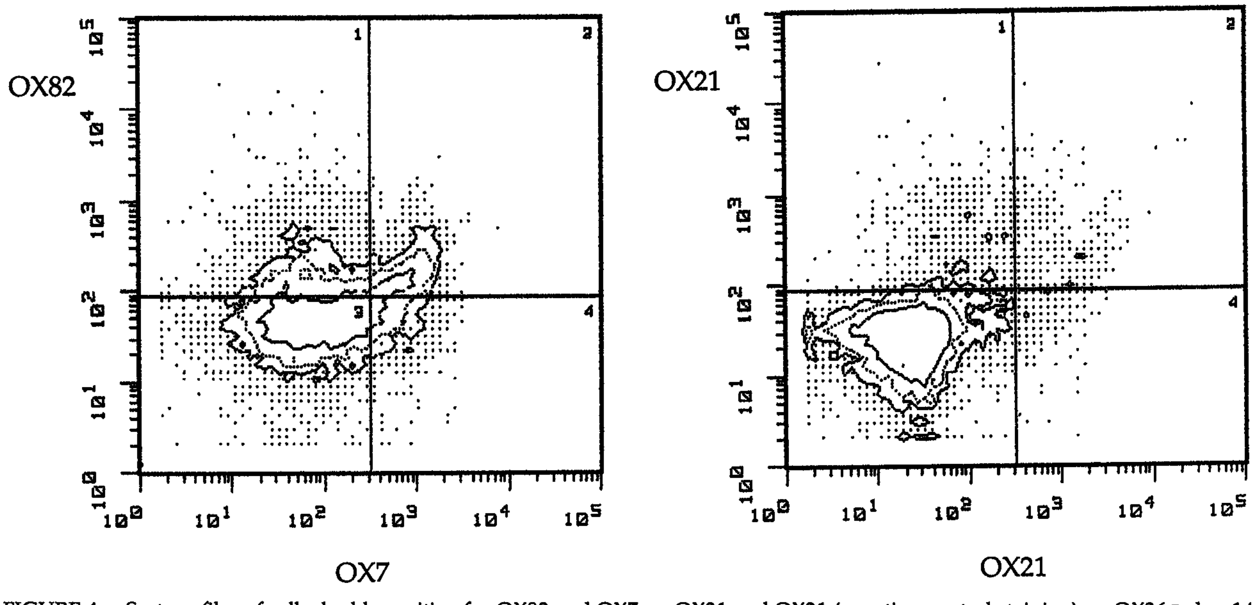

FIGURE 4. Sort profiles of cells double positive for OX82 and OX7, or OX21 and OX21 (negative-control staining) on $OX26^-$ day-14 fetal-liver cells. Double positives were separated from all other cells by the sort gates indicated by the markers. Cells falling into the upper right quadrant were 13% and 3.5% for positive- and negative-control sorts, respectively.

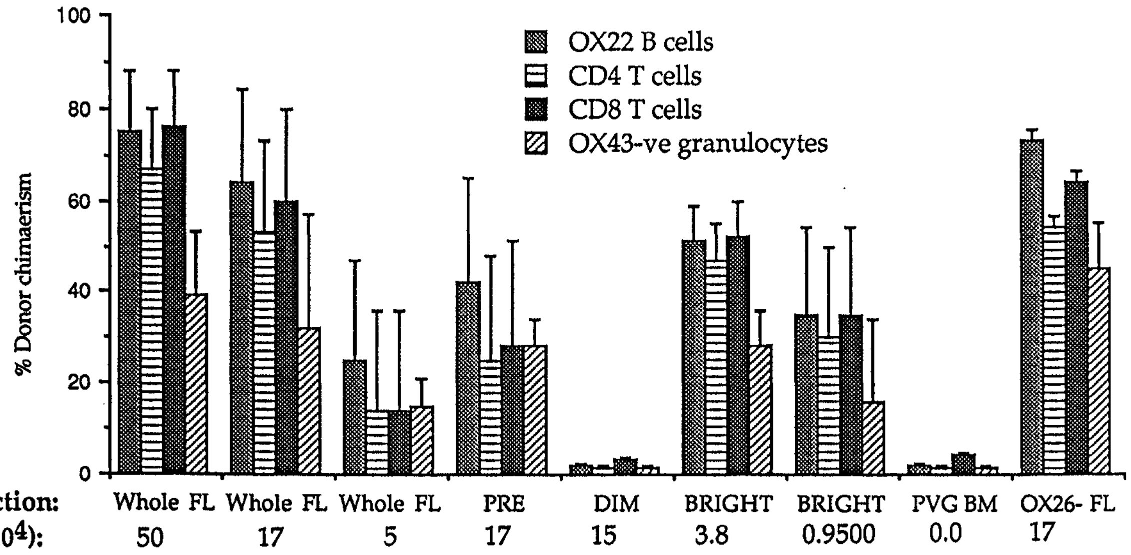

FIGURE 5. Donor-derived chimaerism in peripheral lymph nodes and peritoneal exudate at 10 months following reconstitution with various fractions of day-14 fetal liver having undergone different purification steps. Notation of fractions as Fig. 3(a). "PRE" refers to cells that were rosetted to remove OX26-reactive cells and that were exposed to OX7 and OX82 antibodies but were not sorted. "Dim" are all the sorted cells in the L-shaped segment that were not OX7-OX82 double positives; "Bright" are the sorted cells that were OX7$^+$OX82$^+$. For chimaera analysis, lymph-node cells were stained with OX22 (CD45RC-bright on B cells), OX8 (CD8 on Tc cells), and W3/25 (CD4 on Th cells). Peritoneal cells were stained with OX43 and negative cells designated as neutrophil granulocytes. The indicated numbers of cells injected for each fraction were calculated as described in Materials and Methods. Error bars represent one standard deviation. Two to three recipient rats per group.

two-step purification procedure, we have both enriched the population of presumptive pluripotent stem cells within day-14 fetal liver by up to about 200-fold, and demonstrated for the first time the expression of these three markers on hemopoietic tissue at such an early stage in gestation. Cells were first rosetted to remove almost all of the OX26$^+$ cells, and then FACS sorted to purify the OX82$^+$ OX7$^+$ cells among the remaining OX26-negative cells. Stem-cell activity was measured by the degree of hemopoietic repopulation in radiation chimaeras where host and donor cells express allelomorphically distinct forms of the rat CD45 molecule (RT7; Newton et al., 1986). The use of this marker system has the important advantages that CD45 is expressed on all mature hemopoietic cells, and that the allelic difference between the two forms appears to be immunologically silent (Bell et al., 1989). Using one monoclonal antibody recognizing the donor form of CD45, and a second recognizing a cell subset-specific marker, we have measured levels of CD45 chimaerism in four of the major hemopoietic compartments (CD$^{4+}$ T lymphocytes and sIg$^+$ B lymphocytes, macrophages, and neutrophils). This is an advance on the assay we have already described for lymphopoietic reconstitution (Crook et al., 1987), and that used by Spangrude et al. (1988). In our current work, percentage chimaerism in each of the four compartments measured was essentially at the same level, suggesting that there had been no preferential enrichment of a stem cell committed to one or other lineage. It is theoretically possible that our procedure enriches several different lineage-committed stem cells (progenitors) in equal proportions, but we regard this as unlikely: Within every experiment (Figs. 3a to 3c and data not shown), repopulation did not vary in any lineage over a period of 1 year following reconstitution. Split chimaerism was not found. If there had been a range of lineage-committed cells within the reconstituting population, the degree of their contribution to each compartment would have varied with time due to their probable different reconstitution kinetics. The steadiness of the chimaerism at all times postrepopulation is more simply interpreted as being due to the action of a single kind of more primitive totipotent stem cell. We conclude that the fetal-liver stem cell giving rise to mature macrophages, neutrophils, and CD4 T cells and B cells has the phenotype OX26$^-$OX82$^+$OX7$^+$.

We describe here for the first time the mAb MRC OX82. This predominantly recognizes neutrophils, as judged by (a) forward and side-scatter flow cytometric profiles of bone marrow and peripheral blood (Highnam, Webster; personal communications), and (b) morphological identification of sorted positive cells from bone marrow. Now, and in comparable studies on bone marrow in which we have demonstrated enrichment of long-term repopulating activity by the OX82-positive fraction (Highnam, unpublished), we show that this surface antigen is present not only on mature cells, but also extends back to presumptive totipotent stem cells. Immunohistological studies have also shown its expression on a variety of stromal cell types in a range of nonhemopoietic tissues (Highnam, unpublished), and Western blotting of Brij-96 bone-marrow lysates shows reactivity on a molecule of about 35 kD (unreduced; data not shown). Further biochemical analyses wil be necessary in order to be able to suggest human or murine counterparts for this molecule.

OX26 is specific for the rat transferrin receptor (CD71), and the significance of the lack of expression of this molecule on primitive stem cells has already been discussed by Jefferies et al. (1985a). OX7 recognizes rat Thy-1 and the expression of this molecule on hemopoietic stem cells from rat and mouse is well established (Goldschneider et al., 1978; Hunt, 1979; Berman and Basch, 1985), making it a logical choice to use in association with other mAbs in stem-cell purifications. Here we demonstrate, by the most reliable assay available for stem cells, namely, long-term repopulation, for the first time that this molecule is also expressed on primitive stem cells from day-14 fetal liver in rats. Purification of stem cells form mouse bone marrow indicated that these had a low expression of the Thy-1 molecule (Spangrude et al., 1988).

It might be fruitful to explore in a three-color system whether the $OX7^+OX82^+OX26^-$ population can be further subdivided. Possible candidates suggest themselves from the present studies. Of the other mAbs we tested that stain a minor subpopulation of $OX26^-$ a fetal liver, OX44 (Paterson et al., 1987a) stains the rat equivalent of the membrane transport protein CD53 (Amiot, 1990; Angelisovà et al., 1990). It was absent from the bone-marrow CFU-S population, though the balance sheet for this sort left some CFUs unaccounted for, so a proportion of marrow stem cells may bear OX44 antigen. MHC Class I molecules have been described by others to be present on murine hemopoietic stem cells (van den Engh et al., 1980; Visser et al., 1984), and although we showed here the presence of Class I (as defined by the mAb OX27) on rat bone-marrow CFU-S (Table 1), several attempts to extend this to long-term reconstituting stem cells from rat fetal liver gave equivocal results (data not shown).

Hemopoietic stem cells derived from fetal liver have long been known to be intrinsically different from their adult bone-marrow counterparts. Till et al. (1964) showed that fetal liver could give rise to CFUs with a seemingly unlimited potential for sequential transplantation into new recipients, whereas bone-marrow-derived CFUs were only transplantable a few times until no new CFUs were seen. In addition, fetal-liver-derived CFUs appeared to contain a higher proportion of morphologically undifferentiated cells. Subsequent studies looking at competitive repopulation of irradiated recipients by bone-marrow and fetal-liver cells similarly showed a predominance of the latter to reconstitute the hemopoietic compartment (Micklem and Loutit, 1966; Micklem et al., 1972; Hunt and Fowler, 1981). The reasons for these results are unclear, but it has been suggested that the more primitive the state of stem cells, the higher is their potential for self-renewal (consistent with this, yolk sac cells can be serially transplanted for longer than fetal-liver cells; Metcalf and Moore, 1971). It is also possible that the fetal stem cells have a superior ability to home to the appropriate hemopoietic microenvironment. The experiments described here confirm these findings, in that a dose of undepleted fetal-liver cells containing 10 times fewer cells than a dose of bone marrow injected at the same time repopulated up to 80% of the hemopoietic compartment after 3 months, and that this remained constant for more than a year. Our purification of fetal-liver stem cells at a very early stage of development should assist in characterizing their developmental programming.

### MATERIALS AND METHODS

### Animals

PVG $RT7^a$ rats were bred under specific pathogen-free conditions in the MRC Cellular Immunology Unit, and maintained in a "clean" room (fed on irradiated meal and acidified water, and air supply filtered). Congenic PVG $RT7^b$ rats were bred and maintained under conventional conditions. This strain was obtained by backcrossing the $RT7^b$ allele

(as identified by a polyclonal antiserum; Fabre and Morris, 1974) to PVG inbred rats ($RT7^a$) for twelve generations (Bell et al., 1989). Skin grafts from the congenic strain are permanently accepted by normal PVG $RT7^a$ hosts, and T cells from the two strains show no mutual alloreactivity *in vitro* or *in vivo*.

### Fetal Liver Preparation

Day-14 fetuses (vaginal plug as day 0) were removed from the mothers (of PVG $RT7^b$ strain) and washed twice in Dulbecco's modification of Eagle's medium (DMEM), allowing the fetuses fully to exsanguinate. Livers were dissected out under a low-power dissection microscope. Digestion of livers was performed in 0.5 mg/ml collagenase (Type VIII; Sigma) in DMEM (10 livers/1 ml collagenase) for 30 min at 37°C. The mixture was triturated every 10 min to resuspend the larger pieces of tissue that had settled, as well as to assist in breaking up the livers. After digestion, the livers were placed on ice and washed three times in the standard handling medium (Dulbecco's A + B salts; 10 mM sodium azide 0.1% bovine serum albumin).

### Preparation of Lymph-Node and Peritoneal Exudate Cells

Superficial cervical lymph nodes were biopsied from anesthetised rats at various times after reconstitution. The nodes were teased apart with fine forceps, filtered, and washed in handling medium. For peritoneal exudate preparations, rats were injected intraperitoneally with 5 ml of a 3% solution of proteose peptone in phosphate-buffered saline. Sixteen hours later, 5 ml PBS containing 7.5 units/ml of heparin were also injected intraperitoneally. The abdomen was gently massaged and peritoneal exudate removed through a hole cut in the abdomen. This was transferred to a tube with 1 ml of PBS containing 20 units of heparin. Cells were washed in handling medium, as before.

Cells derived from both these sources were prepared for two-color flow cytometry, with one antibody against a surface molecule specific for a hemopoietic cell subset (revealed by a second layer of FITC-conjugated antibody), and the second antibody as biotinylated 8G6.1 (revealed by a second layer of phycoerythrin-avidin) specific for CD45 from the donor-strain rats.

### Antibodies

Most of the monoclonal antibodies came from fusions performed within the MRC Cellular Immunology Unit: OX7 (anti-rat Thy-1; Mason and Williams, 1980), OX12 (anti-rat kappa light chain; Hunt and Fowler, 1981), OX21 (anti-human C3b inactivator, used as a negative control; Hsiung et al., 1982), OX26 (anti-rat transferrin receptor; Jefferies et al., 1984), OX27 (anti-rat MHC Class I; Jefferies et al., 1985b), OX43 (rat macrophage gp85; Robinson et al., 1986), OX44 (rat CD53 equivalent; Paterson et al., 1987a), OX48 (Paterson et al., 1987b), W3/15 (glycophorin; Hunt, unpublished), W3/25 (CD4) (both Williams et al., 1977). All these antibodies were generous gifts of Dr. A.F. Williams, as were the other mAbs used in initial phenotyping of $OX26^-$ fetal-liver cells (OX1, OX2, OX3, OX32, OX34, OX39, OX40, OX41, OX42, OX43, OX45, OX46, OX47, OX50, OX52, and W3/13). OX82 was prepared in our laboratory following immunization of mice with anemic rat bone marrow, and was detected by screening normal bone marrow for staining of cells with light-scatter characteristics of myeloid cells (Highnam, unpublished). Its myeloid specificity has been confirmed on peripheral blood leucocytes (Webster, personal communication), and by sorting peritoneal exudate populations. Dr. M. Newton kindly supplied the anti-$RT7^a$ monoclonal antibody, NDS.58 (Newton et al., 1986). The anti-$RT7^b$ monoclonal antibody, 8G6.1, was a gift of Dr. D.L. Greiner (Goldschneider et al., 1986). UB11 and UB12 were a gift from Dr. T. Fukumoto (Fujikara et al., 1985). The fluorescein-conjugated rabbit $F(ab')_2$ anti-mouse Ig (Fl-RAM) was a kind gift form Dr. D.W. Mason. The phycoerythrin-avidin conjugate was generously supplied by Drs. J. Trenear and B. Roser (Quadrant International, Cambridge).

### Rosette Depletion of OX26-Positive Fetal Liver Cells

Double rosetting (Hunt, 1986) was found to give rise to a higher yield of OX26-negative cells than other methods of purification tried (such as panning or single rosetting), without any loss in purity (data not shown). Sheep red blood cells (Tissue Culture Services, Berkshire) were used 2–3 weeks after bleeding and coated with OX26 IgG using $CrCl_3$. After washing, 1 ml of this preparation was added to 1 ml of fetal-liver cells ($5 \times 10^7$ cells/ml). The mixture was rotated at 1 rpm for 20 min at 4°C before addition of a further 1 ml of SRBC conjugated as before to the second-layer antibody (rabbit anti-mouse Ig), followed by a further 5 min rotation at 4°C. The samples were transferred to ice and rosettes left to settle. Supernatants were removed after 10 min, and rosettes gently resuspended. Three supernatants were collected in this way to recover the maximum number of $OX26^-$ cells. Cells were washed and any contaminating SRBC removed by lysis in isotonic $NH_4Cl$ buffer. Purities were 92±6% (n = 6), and recoveries about 50%. Around $10^6$ cells were recovered from each liver.

### CFUs Assays

Bone-marrow cells from PVG rats were stained with a range of monoclonal antibodies and sorted into positive and negative populations on the FACS. Sorted cells at various doses were injected into irradiated (9.5 Gy at 1 Gy/min, or 12.5 Gy at 0.05 Gy/min) syngeneic rats of 5 weeks in age. Ten to twelve days later, spleens were removed and fixed in Bouin's solution (60% ethanol, 5% acetic acid, 4% formaldehyde, in water) and macroscopic colonies on the surface of the spleen counted.

### Flow Cytometry

A FACS II (Becton Dickinson, Mountain View, CA) upgraded to FACS IV status was used, with Consort 30 acquisition and analysis software. During sorts, cells were collected in a tube containing 10% bovine serum albumin. As collected cells were often too few to count, cell number was estimated from the deflections electronically recorded by the FACS.

### Radiation Chimaeras

PVG rats ($RT7^a$) of 8–16 weeks of age were whole-body irradiated with a dose of 9.5 Gy at about 1 Gy/min in a $^{137}Cs$ Gammacell 40 irradiator (Nordion, formerly Atomic Energy of Canada) at least 4 hr before intravenous injection of cells. Each rat received a constant dose of $5 \times 10^6$ fresh syngeneic $RT7^a$ bone-marrow cells plus $RT7^b$ fetal-liver cells at the same time. For the fetal liver in some experiments, cells doses for assay of unseparated cells were determined by titration: Unmanipulated cells were titrated over three doses, usually at serial three- or fourfold dilutions. Also, in some experiments, cells that had been exposed to antibody treatment but not separated were assayed at one of these doses to determine whether staining itself affected stem-cell activity. For rosetted and sorted fractions, cell doses were chosen to measure activity per fraction (rather than activity per cell) (Hunt, 1986), and were based on numbers of cells recovered after sorting, not on the input cell number. The sum of the live cell yields after separation (e.g., bright plus dull after sorting, either enumerated by direct counting or calculated from the electronically identified deflections on the sorter) determined dose quanta each equal to a particular dose of rosetted but unsorted cells, usually around $5 \times 10^4$. Within an experiment, the stem-cell activity recovered from the purified fractions was not usually as great as the starting material. Stem cells unavoidably lost some activity, particularly during the 4-stage antibody staining and sorting parts of the protocol, and electronic counting of sorted fractions probably overestimated true cell numbers recovered. Sorted bright and dim fractions were injected in doses numerically proportional to their recovery numbers (so that if there were ten-fold more dim than bright, the assay rats receiving dims received 10 times more than rats receiving brights) and related to the dose quantum for rosetted, unsorted cells. The dose appropriate for whole unseparated fetal liver was calculated by the numerical enrichment factor, knowing the numbers of input and of rosette-depleted cells. No allowance was made for losses of stem-cell activity caused by cell manipulations, and activity losses during separation were therefore presumed to afflict equally all separated fractions and not to have a differential impact. Each fraction was normally assayed in triplicate, using three recipient rats.

### ACKNOWLEDGMENTS

We thank Margaret Hughes, Jane Langley, and Jan Chesshyre for technical assistance, and Louise Baldwin and Reg Boone for supervising the FACS. We are also grateful to Quentin Sattentau and Don Mason for critical reading of the manuscript. KC was supported by a Medical Research Council studentship.

*(Received December 14, 1994)*

*(Accepted June 28, 1995)*

### REFERENCES

Amiot M. (1990). Identification and analysis of cDNA clones encoding CD53: A pan-leukocyte antigen related to membrane transport proteins. J. Immunol. **145:** 4322–4325.

Andrews R.G., Singer J.W., and Bernstein I.D. (1990). Human hematopoietic precursors in long-term culture: Single CD34$^+$ cells that lack detectable T cell, B cell, and myeloid cell antigens produce multiple colony-forming cells when cultured with marrow stromal cells. J. Exp. Med. **172:** 355–358.

Angelisová P., Vlcek C., Stefanova I., Lipoldova M., and Horejsi V. (1990). The human leucocyte surface antigen CD53 is a protein structurally similar to the CD37 and MRC OX-44 antigens. Immunogenetics **32:** 281–285.

Bell E.B., Sparshott S.M., Drayson M.T. and Hunt S.V. (1989). The origin of T cells in permanently reconstituted old athymic nude rats. Analysis using chromosome or allotype markers. Immunology **68:** 547–556.

Berman J.W. and Basch R.S. (1985). Thy-1 antigen expression by murine hematopoietic precursor cells. Exp. Hematol. **13:** 1152–1156.

Bertoncello I., Bradley T.R., Hodgson G.S., and Dunlop J.M. (1991). The resolution, enrichment, and organization of normal bone marrow high proliferative potential colony-forming cell subsets on the basis of rhodamine-123 fluorescence. Exp. Hematol. **19:** 174–178.

Crook K., Highnam S.D.M., and Hunt S.V. (1987). Use of the leukocyte-common (RT7) alloantigen in monitoring enrichment of fetal liver lymphopoietic stem cells. Transplant. Proc. **19:** 3153–3154.

Dick J.E., Magli M.C. Huszar D., Phillips R.A., and Bernstein A. (1985). Introduction of a selectable gene into primitive stem cells capable of long-term reconstitution of the hemopoietic system of W/W$^v$ mice. Cell **42:** 71–79.

Fabre J.W., and Morris P.J. (1974). The definition of a lymphocyte-specific alloantigen system in the rat. Tissue Antigens **4:** 238–246.

Flake A.W., Harrison M.R., Adzick N.S., and Zaniani E.D. (1986). Transplantation of fetal hematopoietic stem cells in utero: The creation of hematopoietic chimaeras. Science **233:** 776–778.

Fujikara Y., Kuniki H., and Fukumoto T. (1985). Monoclonal antibodies against fetal rat liver cells. Bull. Yamaguchi Med. School **32:** 21–26.

Goldschneider I., Gordon L.K., and Morris R.J. (1978). Demonstration of Thy-1 antigen on pluripotent hemopoietic stem cells in the rat. J. Exp. Med. **148:** 1351–1366.

Goldschneider I., Komschlies K.L., and Greiner D.L. (1986). Studies of thymopoiesis in rats and mice. I. Kinetics of appearance of thymocytes using a direct intrathymic adoptive transfer assay for thymocyte precursors. *J. Exp. Med.* **163:** 1–17.

Harrison D.E. (1980). Competitive repopulation: A new assay for long-term stem cell functional capacity. Blood **55:** 77–81.

Hsiung, L.-M., Barclay A.N., Brandon M.R., Sim E., and Porter R.R. (1982). Purification of human C3b inactivator by monoclonal-antibody affinity chromatography. Biochem J. **203:** 293–298.

Hunt S.V. (1979). The presence of Thy-1 on the surface of rat lymphoid stem cells and colony-forming units. Eur. J. Immunol. **9:** 853–859.

Hunt S.V. (1986). Preparative immunoselection of lymphocyte populations. In: Handbook of experimental immunology, 4th ed. Weir, D.M., Blackwell C., Herzenberg L.A. and Herzenberg L.A., Eds. Oxford: Eds. Blackwell Scientific Publications, pp. 55.1–55.18.

Hunt S.V., and Fowler M.H. (1981). A repopulation assay for B and T lymphocyte stem cells employing radiation chimaeras. Cell Tissue Kinet. **14:** 445–464.

Jefferies, W.A. Brandon M.R., Hunt S.V., Williams A.F., Gatter K.C., and Mason D.Y. (1984). Transferrin receptor on endothelium of brain capillaries. Nature **312:** 162–163.

Jefferies W.A., Brandon M.R., Williams A.F., and Hunt S.V. (1985a). Analysis of lymphopoietic stem cells with a monoclonal antibody to the rat transferrin receptor. Immunology **54:** 333–341.

Jefferies, W.A., Green J.R., and Williams A.F. (1985b). Authentic T helper CD4 (W3/25) antigen on rat peritoneal macrophages. J. Exp. Med. **162:** 117–127.

Jones R.J., Wagner J.E., Celano P., Zicha M.S., and Sharkis S.J. (1990). Separation of pluripotent haematopoietic stem cells from spleen colony-forming cells. Nature **347:** 188–189.

Jordan C.T., McKearn J.P., and Lemischka I.R. (1990). Cellular and developmental properties of fetal hematopoietic stem cells. Cell **61:** 953–963.

Keller G. (1992). Clonal analysis of hematopoietic stem cell development *in vivo* Curr. Top. Microbiol. Immunobiol. **177:** 41–57.

Keller G. and Snodgrass R. (1990). Life span of multipotential hematopoietic stem cells *in vivo* J. Exp. Med. **171:** 1407–1418.

Lemischka I.R., Raulet D.R., and Mulligan R.C. (1986). Developmental potential and dynamic behavior of hematopoietic stem cells. Cell **45:** 917–927.

Loken M.R., Shah V.O., Dattilio K.L., and Civin C.I. (1987). Flow cytometric analysis of human bone marrow. I. Normal erythroid development. Blood **69:** 255–263.

Mason D.W., and Williams A.F. (1980). The kinetics of antibody binding to membrane antigens in solution and at the cell surface. Biochem. J. **187:** 1–20.

Metcalf D., and Moore M.A.S. (1971). Haemopoietic Cells. Front. Biol. **24:** 000–000.

Micklem H.S., Ford C.E., Evans E.P., Ogden D.A., and Papworth D.S. (1972). Competitive in vivo proliferation of foetal and adult haematopoietic cells in lethally irradiated mice. J. Cell. Physiol. **79:** 293–298.

Micklem H.S., and Loutit J.F. (1966). In Tissue grafting and radiation (New York: Academic Press), pp. 83–85.

Newton M.R., Wood K.J., and Fabre J.W. (1986). A monoclonal alloantibody detecting a polymorphism of the rat leucocyte common (LC) antigen. J. Immunogenet. **13:** 41–50.

Paterson D.J., Green J.R., Jefferies W.A., Puklavec M., and Williams A.F. (1987a). The MRC OX-44 antigen marks a functionally relevant subset among rat thymocytes. J. Exp. Med. **165:** 1–13.

Paterson D.J., Jefferies W.A., Green J.R., Brandon M.R., Corthesy P., Puklavec M., and Williams A.F. (1987b). Antigens of activated rat T lymphocytes including a molecular of 50,000 Mr detected only on CD4 positive T blasts. Mol. Immunol. **24:** 1281–1290.

Robinson A.P., White T.M., and Mason D.W. (1986). MRC OX-43: A monoclonal antibody which reacts with all vascular endothelium in the rat except that of brain capillaries. Immunology **57:** 231–237.

Spangrude G.J. (1994). Biological and clinical aspects of hematopoietic stem cells. Ann. Rev. Med. **45:** 93–104.

Spangrude G.J., Heimfeld S., and Weissman I.L. (1988). Purification and characterization of mouse hematopoietic stem cells. Science **241:** 58–62.

Srour E.F., Leemhuis T., Brandt J.E., van Besien K., and Hoffman R. (1991). Simultaneous use of rhodamine 123, phycoerythrin, Texas Red, and allophycocyanin for the isolation of human hematopoietic progenitor cells. Cytometry **12:** 179–183.

Szilvassy S.J., Lansdorp P.M., Humphries R.K., Eaves A.C., and Eaves C.J. (1989). Isolation in a single step of a highly enriched murine hematopoietic stem cell population with competitive long-term repopulating ability. Blood **74:** 930–939.

Terstappen L.W.M.M., Huang S., Safford M., Lansdorp P.M,. and Loken M.R. (1991). Sequential generations of hematopoietic colonies derived from single nonlineage-committed CD34$^+$ CD38$^-$ progenitor cells. Blood **77:** 1218–1227.

Till J.E., McCulloch E.A., and Siminovitch L. (1964). Isolation of variant cell lines during serial transplantation of hematopoietic cells derived from fetal liver. J. Nat. Cancer Inst. **33:** 707–720.

Uchida N., Fleming W.H., Alpern E.J., and Weissman I.L. (1993). Heterogeneity of stem cells. Cur. Opin. Immunol. **5:** 177–184.

Van den Engh G.J., Visser J.W.M., Bol S., and Trask B. (1980). Concentration of hemopoietic stem cells using a light-activated cell sorter. Blood Cells **6:** 609–623.

Visser J.W.M., Bauman J.G., Mulder A.H., Eliason J.F., and de Leeuw A.M. (1984). Isolation of murine pluripotent hematopoietic stem cells. J. Exp. Med. **159:** 1576–1590.

Williams A.F., Galfre G., and Milstein C. (1977). Analysis of cell surfaces by xenogeneic myelomahybrid antibodies: Differentiation antigens of rat lymphocytes. Cell **12:** 663–673.

